# Gender differences in grant and personnel award funding rates at the Canadian Institutes of Health Research based on research content area: A retrospective analysis

**DOI:** 10.1371/journal.pmed.1002935

**Published:** 2019-10-15

**Authors:** Karen E. A. Burns, Sharon E. Straus, Kuan Liu, Leena Rizvi, Gordon Guyatt

**Affiliations:** 1 The Li Ka Shing Knowledge Institute, St. Michael’s Hospital, Toronto, Ontario, Canada; 2 The Interdepartmental Division of Critical Care, Department of Medicine, University of Toronto, Toronto, Ontario, Canada; 3 Health Research Methods, Evidence and Impact, McMaster University, Hamilton, Ontario, Canada; 4 Division of General Internal Medicine, Department of Medicine, University of Toronto, Toronto, Ontario, Canada; 5 Dalla Lana School of Public Health, University of Toronto, Toronto, Ontario, Canada; Charité, GERMANY

## Abstract

**Background:**

Although women at all career stages are more likely to leave academia than men, early-career women are a particularly high-risk group. Research supports that women are less likely than men to receive research funding; however, whether funding success rates vary based on research content is unknown. We addressed gender differences in funding success rates for applications directed to one or more of 13 institutes, representing research communities, over a 15-year period.

**Methods and findings:**

We retrospectively reviewed 55,700 grant and 4,087 personnel award applications submitted to the Canadian Institutes of Health Research. We analyzed application success rates according to gender and the primary institute selected by applicants, pooled gender differences in success rates using random effects models, and fitted Poisson regression models to assess the effects of gender, time, and institute. We noted variable success rates among grant applications directed to selected institutes and declining success rates over time. Women submitted 31.1% and 44.7% of grant and personnel award applications, respectively. In the pooled estimate, women had significantly lower grant success (risk ratio [RR] 0.89, 95% confidence interval [CI] 0.84–0.94; *p* < 0.001; absolute difference 3.2%) compared with men, with substantial heterogeneity (I^2^ = 58%). Compared with men, women who directed grants to the Institutes of Cancer Research (RR 0.86, 95% CI 0.78–0.96), Circulatory and Respiratory Health (RR 0.74, 95% CI 0.66–0.84), Health Services and Policy Research (RR 0.78, 95% CI 0.68–0.90), and Musculoskeletal Health and Arthritis (RR 0.80, 95% CI 0.69–0.93) were significantly less likely to be funded, and those who directed grants to the Institute of Aboriginal People’s Health (RR 1.67, 95% CI 1.0–2.7) were more likely to be funded. Overall, women also had significantly lower personnel award success (RR 0.75, 95% CI 0.65–0.86; *p* < 0.001; absolute difference 6.6%). Regression modelling identified that the effect of gender on grant success rates differed by institute and not time. Study limitations include use of institutes as a surrogate identifier, variability in designation of primary institute, and lack of access to metrics reflecting applicants, coapplicants, peer reviewers, and the peer-review process.

**Conclusions:**

Gender disparity existed overall in grant and personnel award success rates, especially for grants directed to selected research communities. Funding agencies should monitor for gender differences in grant success rates overall and by research content.

## Abstract 2

Karen Burns and colleagues reveal the disparity in grant funding between men and women at Canadian research institutes.

## Author summary

**Why was this study done?:**

- Research supports that woman applicants are less likely to receive research funding than men.
- Women at all career stages are more likely to leave academia than men, especially women who are postdoctoral trainees and early in their careers.
- We retrospectively reviewed grant and personnel award submissions based at the Canadian Institutes of Health Research based on research content area to assess for gender differences in funding success rates, which are important to identify whether gender disparity existed in funding. This information is important to address potential sources of bias in the review process.

**What did the researchers do and find?:**

- We retrospectively reviewed 55,700 grant and 4,087 personnel award applications submitted to the Canadian Institutes of Health Research over a 15-year period.
- We found that compared with men, women were significantly less likely to be awarded grants and New Investigator personnel awards.
- Additionally, we identified significant differences in the gradients of grant success in specific research content areas including Cancer, Circulatory and Respiratory Health, Health Services and Policy Research, and Musculoskeletal Health and Arthritis favoring men scientists.
- Regression modelling identified that the effect of gender on grant success rates differed by research community and not time.

**What do these findings mean?:**

- These findings call for heightened awareness in applicants, peer reviewers, and funding agencies to the potential for bias to exist in the evaluation of grant and personnel award applications.
- Our findings highlight the need for funding agencies to monitor gender differences in grant success rates overall and by research content area and to explore possible explanations for gender disparity when identified.
- Additional research is urgently needed to explicate the reasons for gender differences in success rates and identify bias-enhancing conditions in the peer-review process.

## Introduction

In 2000, the Canadian Medical Research Council and the National Health Research Grant Program were merged to form the Canadian Institutes of Health Research (CIHR), comprising 13 institutes [1–3]. Each institute focuses on a specific scientific healthcare domain (e.g., cancer) and includes biomedical, clinical, health services, and policy research, as well as research addressing the health of populations, including societal, cultural, and environmental determinants of health [4]. Under the guidance of the governing council, scientific directors, and institute advisory boards, each institute forges a health research agenda that reflects the health needs of Canadians, the evolution of the Canadian healthcare system, gaps in science, and the information needs of policy decision makers [5].

In the previous funding scheme at the CIHR, research funding was offered through the Open Operating Grant Program (OOGP) and as strategic funding to conduct research in priority areas identified by institute strategic plans in an approximate 70:30 ratio [6]. Through a well-established peer-review process conducted by various committees with content and methodologic expertise, funding was allocated to address gaps in knowledge [1–3,7,8]. To cultivate the development of scientists, peer-review committees at the CIHR also reviewed and funded New Investigator personnel award applications [9].

Several studies provide evidence that women are less likely than men to receive research funding. A study of grant applications submitted to the CIHR open and strategic grant competitions between 2001 and 2011 by scientists who directed their research to the Institute of Health Services and Policy Research found that men under 45 years old were significantly more likely to be funded than age-matched women (odds ratio [OR] 1.40, 95% confidence interval [CI] 1.01–1.95) [4]. Gender differences in success rates have persisted, with female mid- and senior-career researchers being less likely to secure Foundation Scheme (7-year research program funding) in the second pilot competition of the new CIHR funding scheme [10]. Similarly, studies evaluating grant success at the National Institutes of Health (NIH) have shown significant gender disparity in funding success, favoring men in the rate of receipt of initial Research Project Grants (RO1) awards [11], number of career RO1 awards [12], and in productivity as measured by publications and self-perceived success [13]. Although women at all career stages are more likely to leave academia than men, women who are postdoctoral trainees and early in their careers are recognized to be high-risk groups [14–16].

Although institutes do not directly review applications at the CIHR, they reflect scientific communities. Scientists with expertise in content areas are affiliated with particular institutes and participate in peer-review activities related to their context knowledge and expertise. Investigators have not previously addressed the extent to which gender differences have existed in peer-reviewed funding across research communities reflecting research content areas. To examine whether gender differences existed overall in grant and personnel award funding success rates and whether gender differences in success rates differed across research communities, we summarized OOGP grant and personnel award applications approved for funding at the CIHR over a 15-year period in the previous funding scheme.

## Methods

### Study design and population

We retrospectively analyzed data to summarize the number of applications submitted by scientists and scientists in training to the spring and fall OOGP competitions and the New Investigator personnel award competition between 2000/2001 and 2014/2015 according to the self-reported gender and institute selected by the nominated principal investigator (NPI). We excluded bridge grants, priority announcements, and grants that were withdrawn after submission.

### Application process

At the time grants and personnel award applications were submitted, NPIs self-reported their gender and selected one or more primary institute(s) and a peer-review committee reflecting the content of their application. Success rates were based on competition decisions.

### Data sources

After reviewing our brief study proposal (S1 Text), analysts at the CIHR provided data tables in separate, deidentified files for the OOGP grants and the New Investigator Award competitions in Excel (Microsoft Corporation, Redmond, WA, United States) format. Each line in the databases represented a single application and included the year the application was submitted, gender of the NPI, primary institute selected, and funding outcome.

### Ethics

Data were collected by CIHR and held internally by staff at the CIHR as a national funding agency. Research and analytical studies at the CIHR fall under the Canadian Tri-Council Policy Statement 2: Ethical Conduct for Research Involving Humans (TCPS-2) (available from: http://pre.ethics.gc.ca/eng/policy-politique_tcps2-eptc2_2018.html, accessed September 27, 2019). This study had the objective of evaluating gender differences in CIHR funding and falls under Article 2.5 of TCPS-2 and not within the scope of Research Ethics Board review in Canada.

### Statistical analysis

We used descriptive statistics (mean, median, standard deviation, interquartile range, range, and counts) to summarize the number of grant and award applications submitted and approved for funding and to depict success rates (%) for the OOGP and New Investigator Award competitions. We examined success rates by competition, self-reported gender, year, and the primary institute selected by the applicant. Individual applications represented the unit of analysis.

By necessity, we limited analyses pertaining to gender differences in success rates and regression analysis to applications in which the NPI specified his/her gender. We pooled gender differences in relative success rates using random effects models. We derived summary estimates using risk ratios (RRs) across research communities with 95% CIs using Review Manager 5.3 (Cochrane Collaboration, Oxford, United Kingdom) and computed absolute differences from the pooled overall success rates (men–women) over the 15-year period [17]. We evaluated the impact of statistical heterogeneity among pooled studies for each outcome using the I^2^ measure, with threshold values of 0%–40%, 30%–60%, 50%–90%, and ≥75% representing heterogeneity that might not be important or represent moderate, substantial, or considerable heterogeneity, respectively [18,19].

To assess the adjusted relative effects on success rates by gender, time, institute affiliation, and interaction terms (gender and institute, and gender and time) post hoc, we fitted 3 Poisson regression models using robust variance estimators with the observed number of applications awarded (response variable) and the number of applications received by the CIHR [20]. The first model included both interaction terms, the second model included one interaction term (gender and institute), and the third model included no interaction terms. We used the likelihood ratio test to assess the significance of the interaction terms on model fit by comparing 2 sets of nested Poisson regression models (first and second models; second and third models).

## Results

### Grant applications

**Fig 1:**
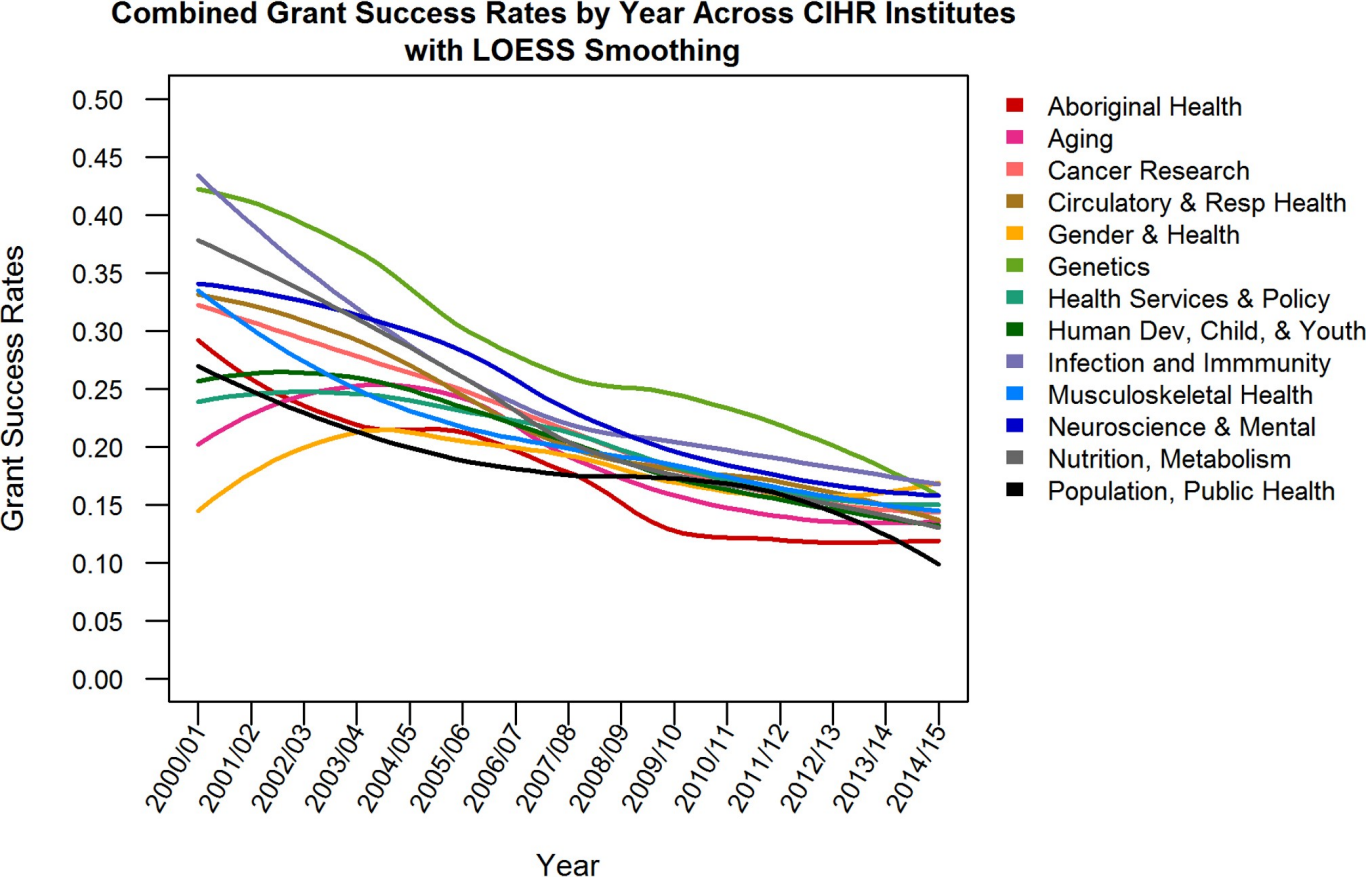
Combined grant success rates by year across CIHR institutes. CIHR, Canadian Institutes of Health Research; LOESS, locally estimated scatterplot smoothing; SD, standard deviation.

Table 1 presents a summary of the number of OOGP applications received, the number approved for funding, and success rates according to institute. The largest number of applications (on average more than 300 grant applications per year) were directed by scientists to the Institutes of Cancer Research, Circulatory and Respiratory Health, Genetics, Infection and Immunity, Neurosciences/Mental Health and Addiction, and Nutrition/Metabolism and Diabetes. Average success rates were lowest among scientists who directed applications to smaller-volume institutes including the Institutes of Aboriginal Peoples’ Health (17.5%), Gender and Health (18.1%), and Population and Public Health (18.6%) and highest among scientists who directed applications to higher-volume institutes such as Genetics (28.7%), Infection and Immunity (25.5%), and Neurosciences/Mental Health and Addiction (24.3%). There was considerable variation in application volume and success rates over time and across designated institutes (Table 1). Success rates in the OOGP decreased over time across institutes except for the Gender and Health institute, which increased after being launched in 2000/2001 and remained relatively stable over time (Figs 1 and S1).

**Table 1:** Number of grant applications received, number approved for funding, and success rate by institute.

CIHR Institutes	Total No. (Range) Applications Received	Average No. Applications Per Year (Mean, SD)	No. Approved for Funding (Median, IQR)	Average Success Rate (%) (Mean, SD)	Success Rate (%) (Range)
Aboriginal Peoples’ Health	426 (10–55)	28.4 (14.3)	4 (2.5, 5.5)	17.46 (7.25)	(9.1–33.3)
Aging	2,854 (71–309)	190 (66.9)	37 (30.5, 40.5)	19.29 (4.89)	(12.3–28.2)
Cancer Research	7,140 (292–655)	476 (121)	102 (93.0, 108.5)	21.98 (6.42)	(14.0–33.9)
Circulatory and Respiratory Health	7,107 (321–682)	474 (101)	102 (97.0, 117.5)	22.64 (7.16)	(13.1–37.3)
Gender and Health	1,270 (36–125)	84.7 (30.4)	16 (13, 19)	18.13 (3.51)	(11.1–23.6)
Genetics	4,566 (164–414)	304 (72.5)	87 (71.0, 93.5)	28.72 (9.50)	(15.9–39.0
Health Services and Policy Research	3,087 (106–371)	206 (69.5)	39 (38, 42)	20.41 (4.05)	(14.8–25.7)
Human Development, Child, and Youth Health	4,027 (189–417)	268 (63.4)	56 (48, 63)	21.27 (6.17)	(11.9–33.2)
Infection and Immunity	5,303 (220–488)	354 (84.2)	86 (80, 93)	25.49 (8.61)	(15.6–43.2)
Musculoskeletal Health and Arthritis	3,738 (167–315)	249 (49.5)	55 (48.5, 57.5)	21.80 (6.25)	(13.7–33.53)
Neurosciences, Mental Health, and Addiction	9,082 (427–833)	605 (113)	145 (131.5, 164)	24.25 (6.92)	(15.5–35.4)
Nutrition, Metabolism, and Diabetes	4,570 (214–418)	305 (67.6)	67 (62.5, 74)	23.08 (7.89)	(12.8–37.9)
Population and Public Health	2,657 (116–307)	177 (60.1)	31 (26.5, 38)	18.60 (4.80)	(9.4–26.7)

Abbreviations: CIHR, Canadian Institutes of Health Research; No., number

### New investigator award applications

On average, more than 25 New Investigator Award applications per year were directed to 5 institutes including Cancer Research, Circulatory and Respiratory Health, Health Services and Policy Research, Infection and Immunity, and Neurosciences/Mental Health and Addiction. Average success rates in the New Investigator competition were lowest among scientists who directed applications to the Institutes of Aboriginal Peoples’ Health (9.0%) and Musculoskeletal Health and Arthritis (13.7%) and highest in Genetics (28.1%) and Infection and Immunity (26.9%) (S1 Table).

### Sex differences in OOGP success

**Fig 2:**
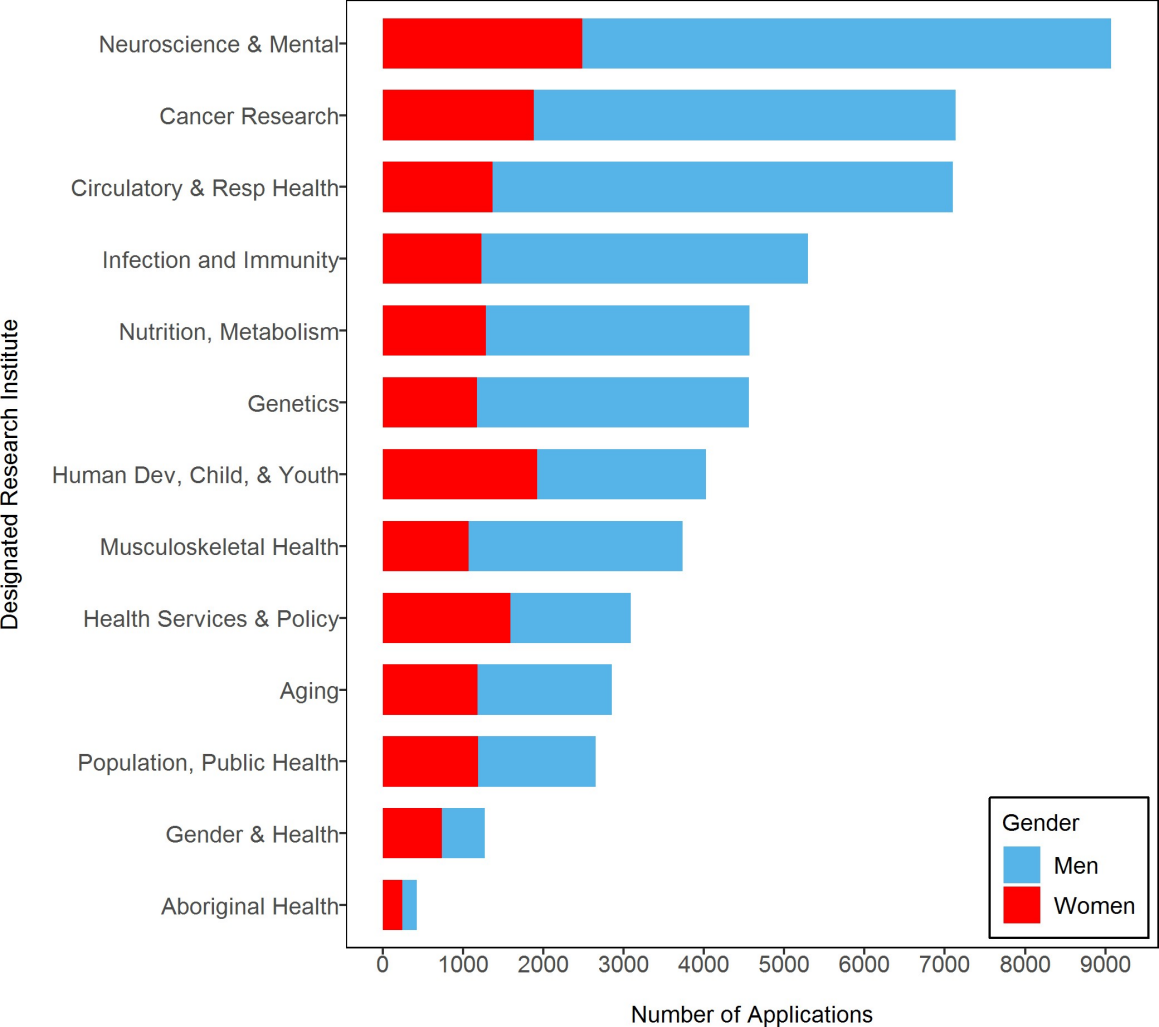
Total number of grant applications submitted by institute and gender.

Fig 2 shows the total number of OOGP applications submitted. Although the number of grant applications submitted by men showed greater variation over time, the number of grant applications submitted by women remained consistently low. Compared with men, however, women submitted more grant applications to 3 institutes: Aboriginal Peoples’ Health (179 versus 245), Gender and Health (533 versus 737), and Health Services and Policy Research (1,496 versus 1,591).

**Fig 3:**
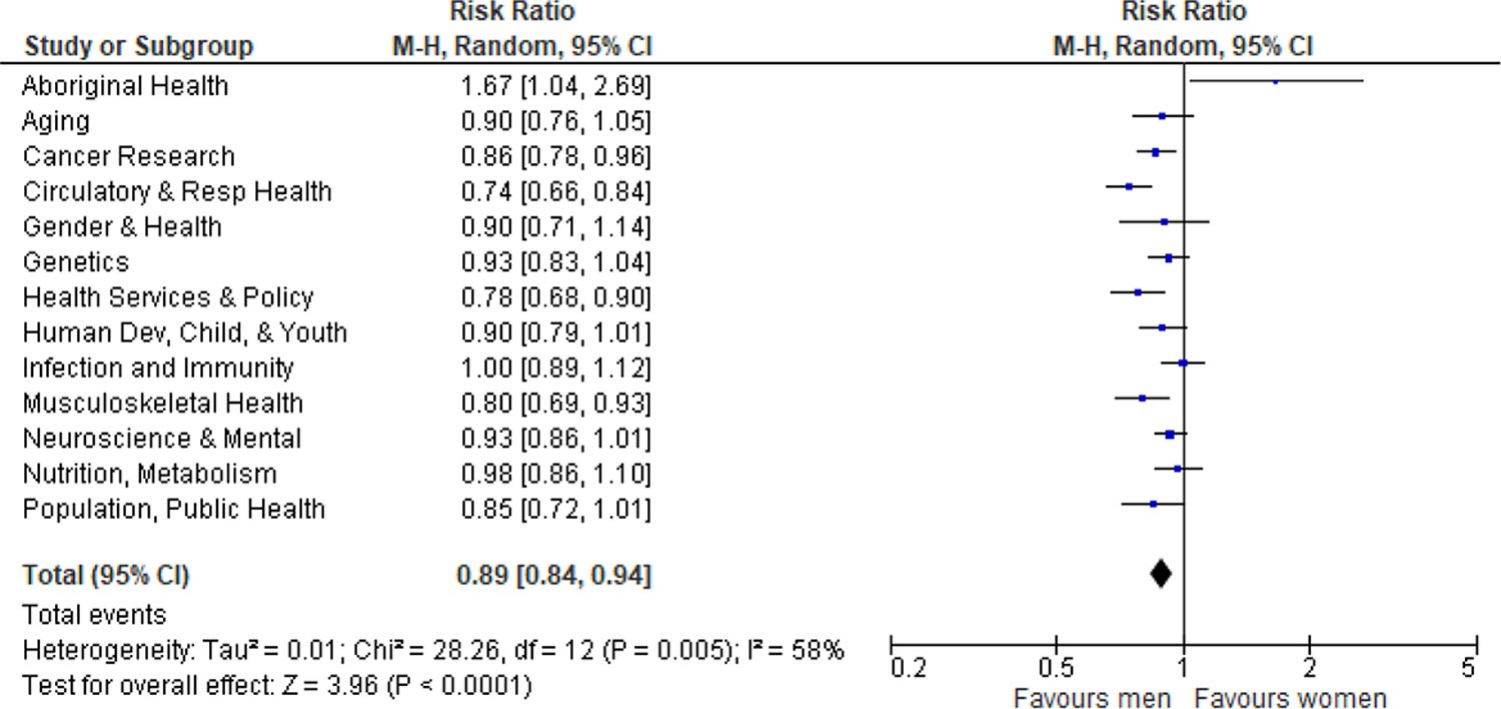
Forest plot of gender differences in grant success rates by designated research institute. CI, confidence interval; M-H, Mantel Haenszel.

In the pooled estimate across institutes, women had significantly lower grant success (RR 0.89, 95% CI 0.84–0.94; *p* < 0.001; absolute difference 3.2%) compared with men, with substantial heterogeneity (I^2^ = 58%). Women who directed applications to the Institutes of Cancer Research (RR 0.86, 95% CI 0.78–0.96), Circulatory and Respiratory Health (RR 0.74, 95% CI 0.66–0.84), Health Services and Policy Research (RR 0.78, 95% CI 0.68–0.90), and Musculoskeletal Health and Arthritis (RR 0.80, 95% CI 0.69–0.93) were significantly less likely to be funded than men. Conversely, women who directed applications to the Institute of Aboriginal Peoples’ Health (RR 1.67, 95% CI 1.0–2.7) were significantly more likely to be funded than men (Fig 3).

### Sex differences in new investigator award success

**Fig 4:**
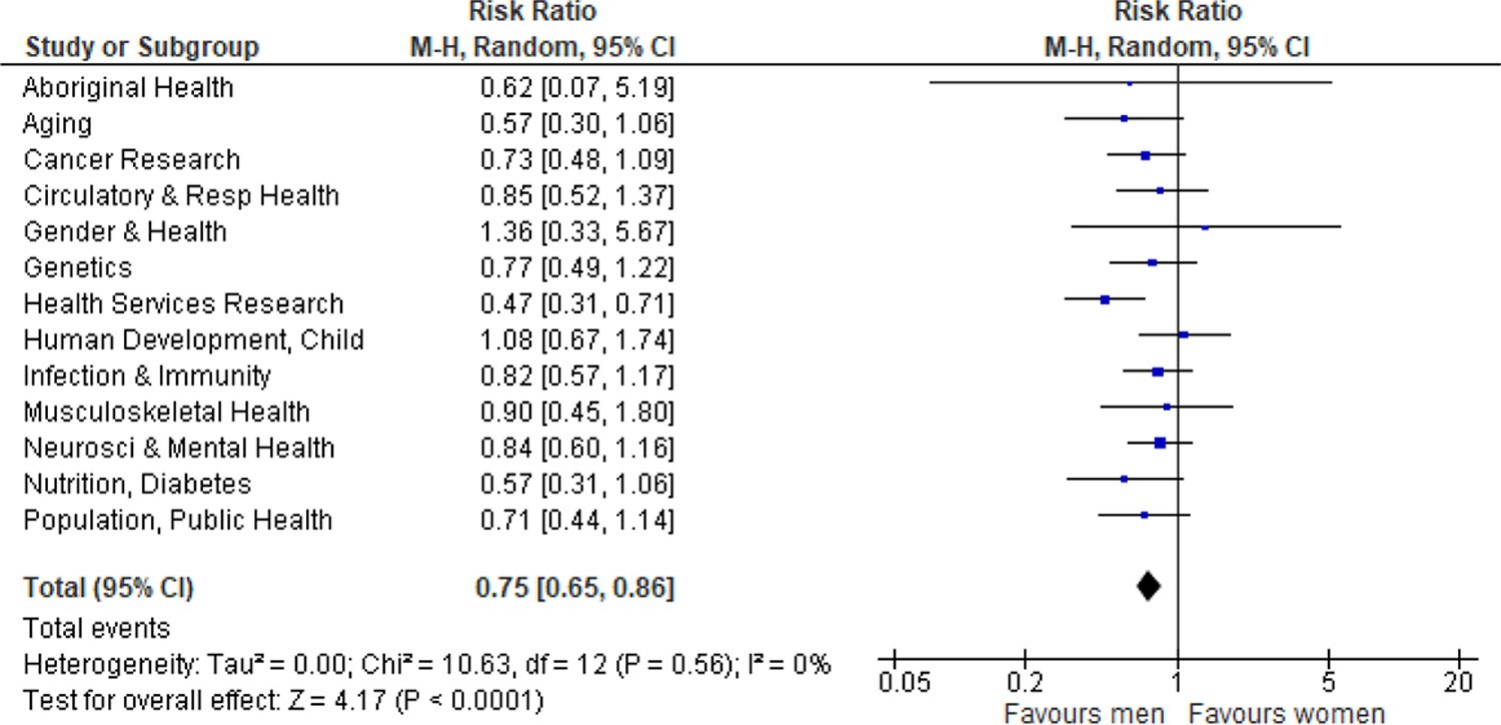
Forest plot of gender differences in new investigator success rates by designated research institute. CI, confidence interval; M-H, Mantel Haenszel.

Compared with men, women directed more New Investigator Award applications to 6 institutes (Aboriginal Peoples’ Health; Aging; Gender and Health; Health Services and Policy Research; Human Development, Child, and Youth; and Population and Public Health). In the pooled estimate, women had significantly lower New Investigator Award success (RR 0.75, 95% CI 0.65–0.86; *p* < 0.001; absolute difference 6.6%) compared with men, with very little heterogeneity (I^2^ = 0%) (Fig 4).

### Poisson regression

Using the likelihood ratio test, we did not identify significant differences (*p* = 0.6) between the first model including both interaction terms (gender and institutes, gender and time) and the second model including only the interaction between gender and institutes, suggesting that removal of the interaction between gender and time does not statistically impact model fit, and the simpler model is preferred. However, the likelihood ratio test between the second model, containing the interaction term between gender and institutes, and the third model, which did not contain interaction terms, was statistically significant (*p* = 0.02), suggesting that the effect of gender on grant success rates differed by designated institute (research content area) and not time.

## Discussion

Over 15 years of funding at the CIHR, we noted variable success rates among grant applications directed to various institutes and declining success rates over time. Overall, women had significantly lower grant success (RR 0.89, 95% CI 0.84–0.94; *p* < 0.001; absolute difference 3.2%) compared with men. Across institutes, we found substantial heterogeneity, with variability demonstrated both in the primary analysis for grant applications and in the Poisson regression. Women who directed grants to 4 institutes (Cancer Research, Circulatory and Respiratory Health, Health Services and Policy Research, and Musculoskeletal Health and Arthritis) were significantly less likely to be funded than men. However, women who directed grants to the Institute of Aboriginal Peoples’ Health were more likely to be funded than men. In pooled estimates, women also had significantly lower personnel award success (RR 0.75, 95% CI 0.65–0.86; *p* < 0.001; absolute difference 6.6%) compared with men, with no heterogeneity. Poisson regression models supported that the effect of gender on grant success rates differed by designated institute and not time.

Our review has several strengths. It is novel in assessing the extent to which gender differences in peer-reviewed funding exist across research communities, represented by CIHR institutes, over a 15-year period in the former funding scheme. We summarized data using both relative and absolute effect measures [21,22] and report our findings using recommendations for cohort studies (S1 Strobe checklist). We identified significant differences in the gradients of grant success in specific research content areas, including Cancer, Circulatory and Respiratory Health, Health Services and Policy Research, and Musculoskeletal Health and Arthritis, favoring men scientists. We found that relative effects of gender were stable over time and, consequently, absolute differences decreased as funding success decreased for both men and women.

Our review also has limitations. First, we analyzed data from the perspective of the applicant. Although applicants did not submit applications directly to CIHR institutes, we used their self-designated research institute as a surrogate identifier to assess whether gender differences existed in grant and personnel award funding overall and across research communities, reflecting research content areas. Notwithstanding, variability may have existed in how NPIs designated a primary institute based on the content of individual grants or award applications. Furthermore, in a small proportion of applications, the primary institute may not have been identified correctly by the NPI or the NPI may have identified more than one institute. Second, we did not have access to metrics that reflected applicant and coapplicant demographics (nationality, race) and quality (training, rank, experience, productivity), peer reviewer demographics and ratings, or features of the peer-review process itself [23,24]. Third, because few grant applications were directed to the Institute of Aboriginal Peoples’ Health, and even fewer personnel award applications were submitted overall, CIs were broad in corresponding summary estimates of effect.

In our study, women submitted 31.1% and 44.7% of grant and personnel award applications, respectively. These findings align with those of other studies conducted in the US and UK, which identified that women submitted fewer grant applications [25,26]. Data from the UK, NIH, Danish Research Council, and the European Research Council indicate that women were less likely than men to secure grant funding [12, 27–29], held fewer large-scale grants [26], and received less funding in both absolute and relative terms [30]. In a similar longitudinal analysis of 34,770 NIH-funded grants, Hechtman and coworkers found that women submitted fewer applications, held fewer grants, and were less likely to have their grants renewed [31].

Compared with women, men have higher training program award success rates [32–35]. Using NIH data, Jagsi and colleagues found that women received significantly fewer Mentored Career Development (K08) (31.4%) and K23 (43.7%) Awards compared with men [13]. Among recipients of career development awards, women were also significantly less likely than men to receive independent grant funding [11]. In their review of 2,823 early-career scientist applications, van der Lee and colleagues found that men had higher funding success rates and received significantly more competitive “quality of researcher” as opposed to “quality of proposal” evaluations [35]. In their study, gender disparities were most prevalent in scientific disciplines with the highest application volume and with similar gender representation [35]. Under these conditions, they hypothesized that reviewers found it challenging to thoroughly process and weigh applications, increasing the chance that they relied on heuristics in formulating their evaluations. Heuristics, in turn, have been associated with implicit bias [36,37]. Finally, through content analysis, van der Lee and colleagues noted the use of “gendered language” in instructional and evaluation materials that may have favored male applicants [35]. Contrary to their findings, we did not find a relationship between application volume and the visibility of woman applicants in either grant or personnel award competitions.

There are 2 potential explanations for the differential success gradients. Either the applications from women are inferior to those of men or bias against women is responsible for the difference. There is no compelling evidence to support that woman and man researchers are not equally capable. A recent study has shown that although differences in “quality of proposal” ratings were not different between grant applications submitted by women and men, women’s applications were awarded significantly lower “quality of researcher” assessments and were significantly less likely to be prioritized compared with those of men [35]. As data reflecting applicants’ track record were not available for analysis, we cannot rule out the possibility that gender disparities in productivity or features related to productivity (age, authorship position, academic rank) [38] resulted in differential applicant evaluations in our study. Although direct comparisons of funding rates between scientific agencies are difficult because of differences in the type of grants awarded and review procedures, at least one feature of the review process has been identified as particularly prone to gender bias—assessments of applicant’s track records of success [39]. Of interest, a gender gap in funding rates has not been demonstrated with NIH RO1 project grants in which reviewers focus solely on the “quality of the proposal.” By contrast, gender disparity disadvantaging women has been demonstrated in funding rates for RO1 renewal and equivalent awards in which applicants’ track records of success were heavily weighted by reviewers [12,40]. Our findings add to this literature by demonstrating gender differences in gradients of grant success in specific research content areas. Compared with man scientists, woman scientists who submit grant applications in these content areas either submit lower-quality grants or are disadvantaged as applicants.

The introduction of review procedures that exclusively focus on the research proposal is a promising mechanism to reduce gender bias in grant reviews. However, the utility of this approach for evaluating personnel award applications, in which productivity has traditionally been a key consideration, is controversial [35,39]. Other forms of bias including intentional or unintentional reviewer bias, unconscious bias, and implicit gender bias may also, individually or collectively, influence the review process [23, 41–43]. To this end, the Swedish Medical Research Council investigators found that woman scientists required 2.5 times as many impactful publications as man scientists to receive an equivalent score for scientific competence by peer reviewers—a finding that has been reproduced in other studies [44–46]. Although authors have proposed several solutions to address the gender disparity in funding, including gender-equal review committees [47,48], mentoring programs [49], gender policy endorsement [35], principal investigator anonymity [50], de-emphasizing researcher quality in grant applications [35,44,50,51], and using gender neutral language [35,52,53], no study has systematically evaluated the reasons for gender differences in grant and personnel award success rates. Without a clear understanding of the reasons for the observed differences in success, the proposed solutions are speculative and may be harmful. For example, repeated emphasis on gender policies to advance women, especially when both genders are equally represented among applicants, may mislead reviewers into thinking that gender bias is not an issue. With this “paradox of equality,” reviewers may be less vigilant for unequal outcomes, thereby increasing the likelihood of “gender imbalance” occurring in the review processes [54,55].

Our data support the call for mandatory reporting of gender in grant and personnel award applications at national funding agencies [56]. Moreover, our findings highlight the need for funding agencies to monitor gender differences in success rates overall and by research content area and explore possible explanations for gender disparity when identified. Additional research is urgently needed to explicate the reasons for gender differences in success rates, overall and by content area, and to identify “bias-enhancing conditions” in the peer-review process.

## Conclusions

Gender disparity existed overall in grant and personnel award success rates, especially for grants directed to selected research communities. Funding agencies should monitor gender differences in grant success rates by content and explore possible explanations for gender disparity when identified.

## Supporting information

S1 Fig Combined grant success rates by year across CIHR institutes (actual data). CIHR, Canadian Institutes of Health Research.
(TIFF)

S1 Text Research proposal. CIHR, Canadian Institutes of Health Research; OOGP, Open Operating Grant Program.
(DOCX)

S1 Table New investigator award applications received and approved for funding and success rate by institute. SD, standard deviation.
(DOCX)

S1 Strobe checklist Checklist of items included in this retrospective review. Para, paragraph.
(DOCX)

## Abbreviations

CI: confidence interval
CIHR: Canadian Institutes of Health Research
LOESS: locally estimated scatterplot smoothing
M-H: Mantel Haenszel
NIH: National Institutes of Health
NPI: nominated principal investigator
OOGP: Open Operating Grant Program
OR: odds ratio
RR: risk ratio
TCPS-2: Tri-Council Policy Statement 2: Ethical Conduct for Research Involving Humans
