# Supplementary figures and images for: Gender differences in grant and personnel award funding rates at the Canadian Institutes of Health Research based on research content area: A retrospective analysis
Source: PLoS Med. 2019 Oct 15;16(10):e1002935. doi: 10.1371/journal.pmed.1002935 (PMC6793847; doi:10.1371/journal.pmed.1002935)

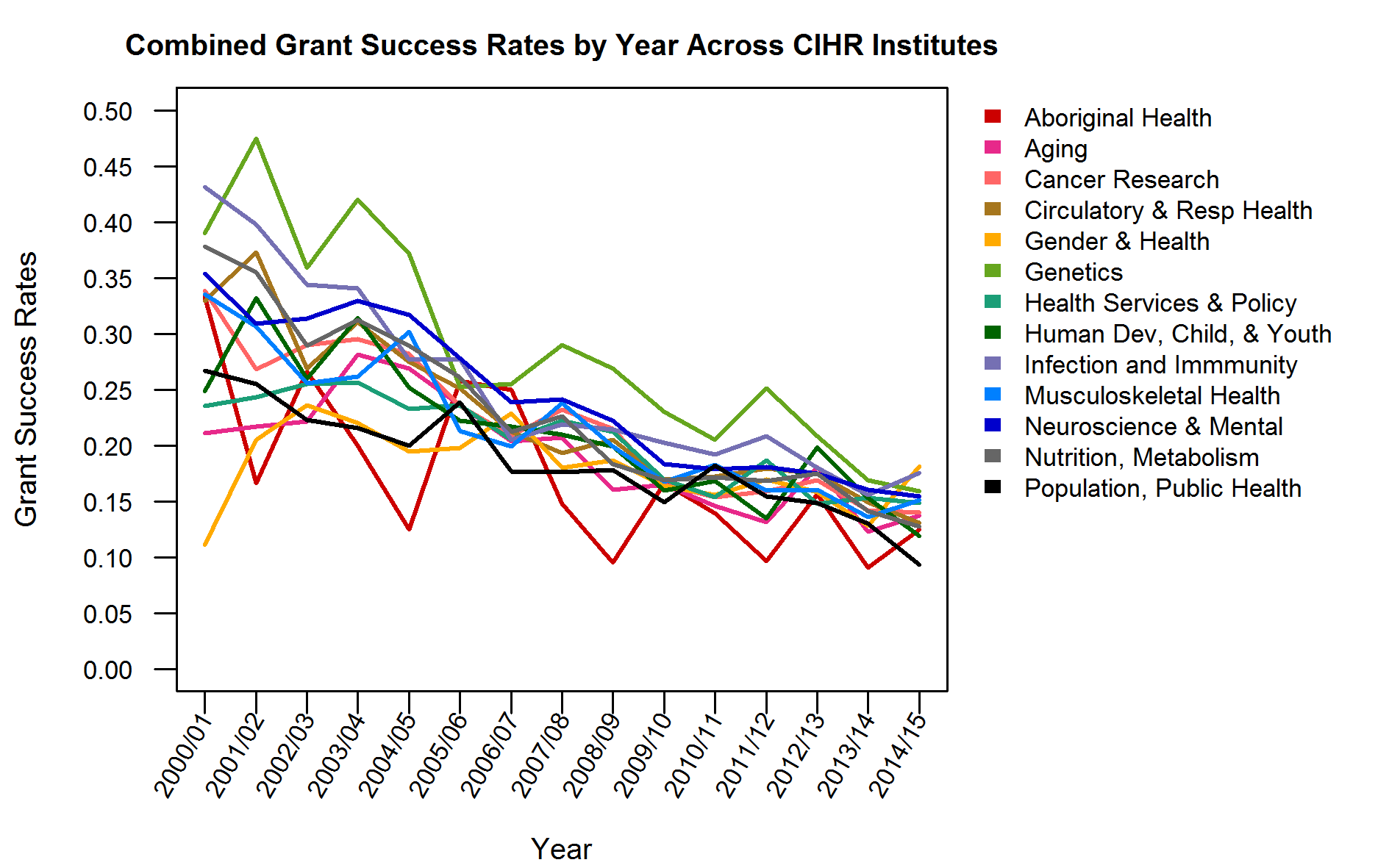

Supplement: S1 Fig — CIHR, Canadian Institutes of Health Research. (TIFF) [file pmed.1002935.s001.tiff]
