# Supplementary material for: Gender differences in grant and personnel award funding rates at the Canadian Institutes of Health Research based on research content area: A retrospective analysis
Source: PLoS Med. 2019 Oct 15;16(10):e1002935. doi: 10.1371/journal.pmed.1002935 (PMC6793847; doi:10.1371/journal.pmed.1002935)
Supplement: S1 Text — CIHR, Canadian Institutes of Health Research; OOGP, Open Operating Grant Program. (DOCX) [file pmed.1002935.s002.docx]

**Within-Sex Differences in Application and Success Rates Across 15 Years of the CIHR**

**Open Operating Grants and Early Career Award Competitions**

**Research Plan: Karen E. A. Burns**

**Date: 17 August 2017**

**NB: Text for two paragraph summary (as requested).**

____________________________________________________________________________________

**Introduction**

In 2000, the Canadian Medical Research Council and the National Health Research Grant Program were merged to form the Canadian Institutes of Health Research (CIHR) [1-3]. The CIHR sought to foster collaborative research through the creation of health research institutes that encompassed funding for biomedical research, clinical and health systems research, and research addressing health services, the health of populations, environmental influences on health, and societal and cultural dimensions of health [4,5]. From 2000/2001 to 2014/2015 most research funding was allocated through the open operating grants program (OOGP). The OOGP aimed to fund high quality science across mandates of the 13 institutes through a well-established peer review process. During this time, CIHR also reviewed and funded applications for personnel awards (i.e., Clinician Scientist Phase 1 & 2 Awards and New Investigator Awards) to cultivate career scientists, foster mentorship, and develop the next generation of scientists in Canada [6].

**Rationale**

The rationale for examining sex differences in grant and early personnel award success rates is three fold:

a) To reflect upon within sex differences in funding using a large national dataset and experience (15 -year funding period) including 13 research institutes representing different fields of interest and the 2 largest salary support programs for early researchers,

**Objectives**

The objectives of the proposed study are to summarize within-sex differences in application and success rates across 15-years of the OOGP and Early Career Award competitions.

**Methods**

Working with CIHR, we will assemble a cohort of all full grant applications submitted to the CIHR OOGP in the spring and fall competitions from 2000/2001 to 2014/2015 and the two most career award competitions (CIHR Clinician Scientist (phase 1 and 2 awards) and the New Investigator awards)

**Statistical Analysis**

Descriptive statistics (mean, median, standard deviation, interquartile range, range) will be used to characterize funding trends between 2000 and 2014, including the proportions funded, funds awarded and success rates. We will tabulate within-sex funding success rates based on self-reported NPI sex where declared. The declared primary institute will be the unit of analysis. We will exclude applications wherein no gender or institute is specified.

To account for differences in the number of grant and career award applicants by sex, we will tabulate within-sex differences and compare them using the Chi-square tests with Yates’ correction for continuity. We will compute within-sex differences in grant success rate by funding year and compute average differences over the 15-year funding period within each institute.

**REFERENCES**

1. Hoey J. Social sciences meet basic sciences in new era of health-services research. *CMAJ* 2001;164:248.

2. Gray C. Clinicians will benefit from new research initiative, CIHR promises. *CMAJ* 2000;163:586.

3. Kondro W. CIHR floats “research-into-action” trial balloon. *CMAJ* 2003; 168:209.

# 4. Canadian Institutes of Health Research Act (S.C. 2000, c. 6). Available at <http://laws.justice.gc.ca/eng/acts/C-18.1/(accessed> August 18, 2017)

5. Tamblyn R, McMahon M, Girard N, Drake E, Nadigel J, Gaudreau K. Health services and policy research in the first decade at the Canadian Institutes of Health Research. CMAJ Open. 2016; 4(2): E213±21. Epub 2016/07/12. doi: 10.9778/cmajo.20150045 PMID: 27398366

6. [Golper TA](https://www.ncbi.nlm.nih.gov/pubmed/?term=Golper%20TA%5BAuthor%5D&cauthor=true&cauthor_uid=18322049)^1^, [Feldman HI](https://www.ncbi.nlm.nih.gov/pubmed/?term=Feldman%20HI%5BAuthor%5D&cauthor=true&cauthor_uid=18322049). New challenges and paradigms for mid-career faculty in academic medical centers: key strategies for success for mid-career medical school faculty. [Clin J Am Soc Nephrol.](https://www.ncbi.nlm.nih.gov/pubmed/18322049) 2008 Nov;3(6):1870-4. doi: 10.2215/CJN.03900907. Epub 2008 Mar 5.
