# Supplementary material for: Gender differences in grant and personnel award funding rates at the Canadian Institutes of Health Research based on research content area: A retrospective analysis
Source: PLoS Med. 2019 Oct 15;16(10):e1002935. doi: 10.1371/journal.pmed.1002935 (PMC6793847; doi:10.1371/journal.pmed.1002935)
Supplement: S1 Table — SD, standard deviation. (DOCX) [file pmed.1002935.s003.docx]

**Supplemental Table 1: New Investigator Award Applications Received, Approved for Funding and Success Rate by Institute**

| **CIHR Institutes** | **Total No. (range)**  **Applications**  **Received** | **Average No. Applications**  **Per Year**  (Mean, SD) | **No. Approved for Funding**  (Median, IQR) | **Average Success Rate (%)**  (Mean, SD) | **Success Rate (%)**  (Range) |
| --- | --- | --- | --- | --- | --- |
| Aboriginal Peoples’ Health | 47 (2-7) | 3.6 (1.5) | 0 (0, 1.0) | 9.0 (16.2) | (0-50.0) |
| Aging | 270 (8-31) | 18.0 (6.7) | 2.0 (1.5, 3.5) | 14.1 (10.1) | (0-37.5) |
| Cancer Research | 404 (18-39) | 26.9 (6.2) | 6.0 (5.0,6.0) | 22.7 (6.48) | (14.3-36.8) |
| Circulatory and Respiratory Health | 404 (16-41) | 26.9 (8.3) | 5.0 (4.0-7.0) | 19.0 (6.3) | (4.5-26.9) |
| Gender and Health | 106 (2-22) | 7.07 (5.48) | 0 (0, 1.0) | 12.5 (18.8) | (0-66.7) |
| Genetics | 252 (6-24) | 16.8 (6.2) | 5.0 (2.5-6.5) | 28.1 (11.7) | (0-41.7) |
| Health Services and Policy Research | 435 (18-47) | 29.0 (7.5) | 5.0 (4.0-6.0) | 17.2 (5.3) | (10.8-32.1) |
| Human Development, Child, and Youth | 343 (14-33) | 22.9 (5.3) | 4.0 (3.0-5.0) | 18.2 (9.4) | (0-35.7) |
| Infection and Immunity | 394 (14-40) | 26.3 (8.3) | 7.0 (4.5-8.5) | 26.9 (7.6) | (14.3-45.45) |
| Musculoskeletal Health and Arthritis | 227 (7-24) | 15.1 (4.9) | 2.0 (1.5 -3.0) | 13.7 (7.5) | (0-25.0) |
| Neurosciences, Mental Health, and Addiction | 629 (19-62) | 41.9 (12.7) | 9.0 (7.0 -10.0) | 21.0 (5.46) | (9.5-30.0) |
| Nutrition, Metabolism, and Diabetes | 281 (13-24) | 18.7 (3.4) | 3.0 (2.0-4.0) | 17.1 (6.6) | (5.9-29.4) |
| Population and Public Health | 299 (8-37) | 19.9 (7.6) | 4.0 (2.0-5.5) | 19.1 (13.0) | (0-36.8) |
